# Supplementary material for: Predicting visual function by interpreting a neuronal wiring diagram
Source: Nature. 2024 Oct 2;634(8032):113–23. doi: 10.1038/s41586-024-07953-5 (PMC11446822; doi:10.1038/s41586-024-07953-5)
Supplement: Supplementary file 6 — CRF and ERF predictions for individual TmY4 and TmY9 cells. Analogous to Supplementary Data 3, but for TmY target types. Shown are the top four monosynaptic pathways, the strongest pathway passing through each of the top ten intermediary types (ranking from Extended Data Fig. 7), and the trisynaptic pathway Tm1–TmY–Dm3–TmY (see the section entitled Prediction of spatial normalization). [file 41586_2024_7953_MOESM6_ESM.zip › DataS4/TmY9qΓèÑ/720575940610903250.pdf]

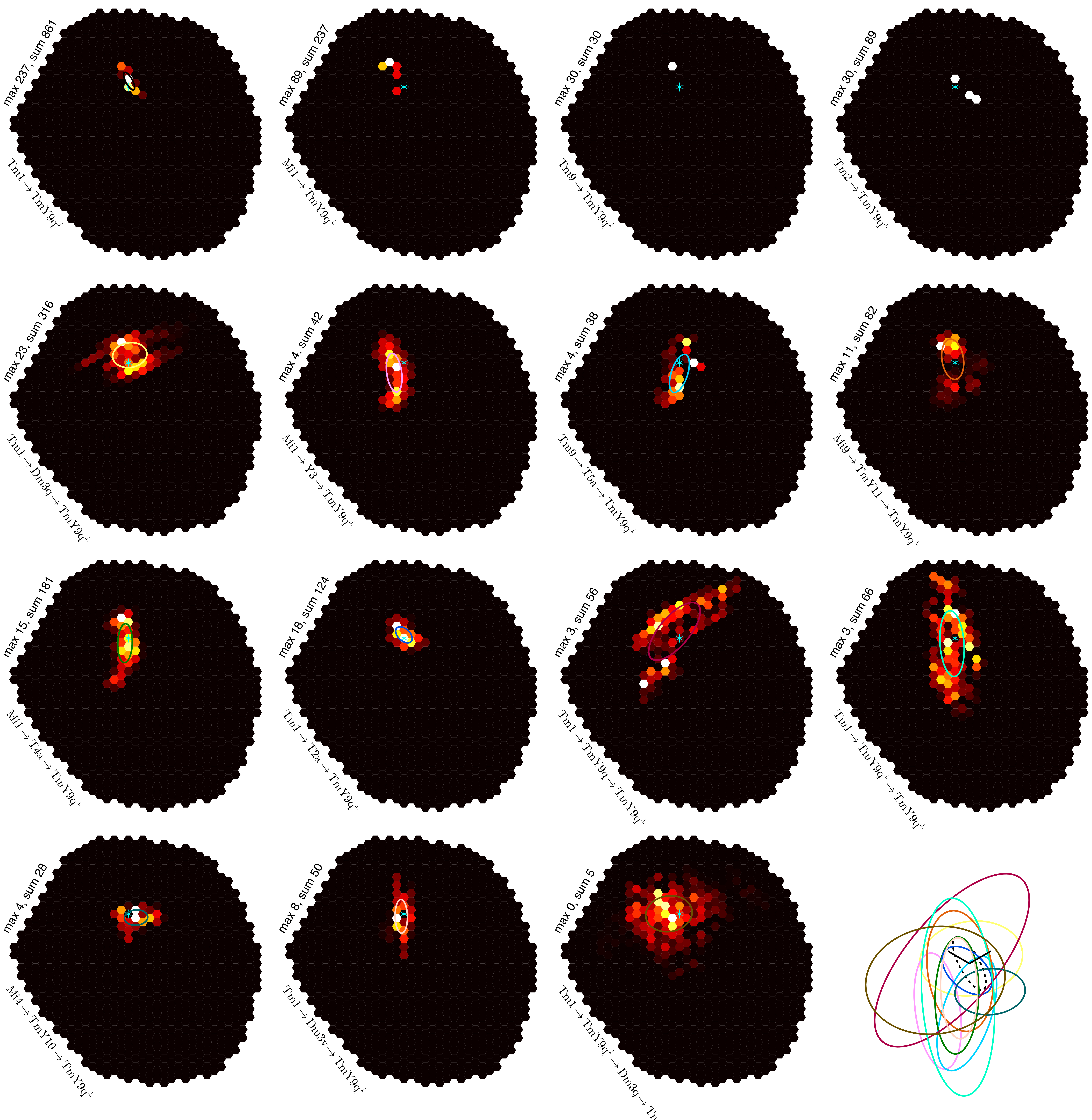
max 237, sum 861
Tm1 → TmY9q⊥
max 89, sum 237
Mi1 → TmY9q⊥
max 30, sum 30
Tm9 → TmY9q⊥
max 30, sum 89
Tm2 → TmY9q⊥
max 23, sum 316
Tm1 → Dm3q → TmY9q⊥
max 4, sum 42
Mi1 → Y3 → TmY9q⊥
max 4, sum 38
Tm9 → T5a → TmY9q⊥
max 11, sum 82
Mi9 → TmY11 → TmY9q⊥
max 15, sum 181
Mi1 → T4a → TmY9q⊥
max 18, sum 124
Tm1 → T2a → TmY9q⊥
max 3, sum 56
Tm1 → TmY9q → TmY9q⊥
max 3, sum 66
Tm1 → TmY9q⊥ → TmY9q⊥
max 4, sum 28
Mi4 → TmY10 → TmY9q⊥
max 8, sum 50
Tm1 → Dm3v → TmY9q⊥
max 0, sum 5
Tm1 → TmY9q⊥ → Dm3q → TmY9q⊥
